# Spatial organization of excitatory synaptic inputs to layer 4 neurons in mouse primary auditory cortex

**DOI:** 10.3389/fncir.2015.00017

**Published:** 2015-04-29

**Authors:** Megan B. Kratz, Paul B. Manis

**Affiliations:** ^1^Department of Otolaryngology/Head and Neck Surgery, University of North Carolina at Chapel HillChapel Hill, NC, USA; ^2^The Curriculum in Neurobiology, University of North CarolinaChapel Hill, NC, USA; ^3^Department of Cell Biology and Physiology, University of North CarolinaChapel Hill, NC, USA

**Keywords:** glutamate uncaging, spectral integration, intracortical circuits, patch clamp, brain slice, neocortical circuits, thalamocortical recipient neurons

## Abstract

Layer 4 (L4) of primary auditory cortex (A1) receives a tonotopically organized projection from the medial geniculate nucleus of the thalamus. However, individual neurons in A1 respond to a wider range of sound frequencies than would be predicted by their thalamic input, which suggests the existence of cross-frequency intracortical networks. We used laser scanning photostimulation and uncaging of glutamate in brain slices of mouse A1 to characterize the spatial organization of intracortical inputs to L4 neurons. Slices were prepared to include the entire tonotopic extent of A1. We find that L4 neurons receive local vertically organized (columnar) excitation from layers 2 through 6 (L6) and horizontally organized excitation primarily from L4 and L6 neurons in regions centered ~300–500 μm caudal and/or rostral to the cell. Excitatory horizontal synaptic connections from layers 2 and 3 were sparse. The origins of horizontal projections from L4 and L6 correspond to regions in the tonotopic map that are approximately an octave away from the target cell location. Such spatially organized lateral connections may contribute to the detection and processing of auditory objects with specific spectral structures.

## Introduction

Sensory systems detect, identify, and track objects in the environment. In the auditory system, these objects often contain component frequencies that are spectrally discontinuous and distributed across most of the hearing range. This presents a problem for auditory processing because reassembling sounds as objects rather than as individual component frequencies requires that some auditory neurons integrate sensory activity over broad spatial ranges, which is not readily accomplished with spatially restricted local circuits. Indeed, most (but not all) pathways in the auditory brainstem and midbrain retain the tonotopically-organized narrow frequency representation that arises in the cochlea. However, previous studies have suggested that extensive cross-frequency excitatory connectivity occurs within the primary auditory cortex (A1), and thus this may be one site in the auditory pathway where such cross-frequency convergence is necessary to begin to assemble auditory objects from the neural representation of sound. However, the organization of these circuits is not well understood.

Evidence for circuits that can support integration across frequency in A1 comes from multiple studies. Neurons in A1 have subthreshold tuning curves that are broader than can be accounted for by their thalamic inputs (Kaur et al., 2004, 2005; Happel et al., 2010). When intracortical A1 activity is silenced by the injection of the GABA agonist muscimol into A1, intracellularly recorded subthreshold tuning curves become narrower, reflecting the tuning of medial geniculate neurons that project to cortex (Kaur et al., 2004). Tonal stimuli at the characteristic frequency generate shorter latency current sinks in the extracellular space than stimuli at other frequencies, suggesting that the off-frequency excitatory connections are processed through additional intracortical circuits (Kaur et al., 2005). *In vivo* calcium imaging has shown that even adjacent spines on individual layer 2/3 (L2/3) pyramidal cells in A1 can be tuned to widely different frequencies (Chen et al., 2011), indicating that individual L2/3 cells receive convergent input from different portions of the acoustic spectrum, although the sources of these inputs are unclear. Functional mapping of intracortical circuits in A1 using glutamate uncaging has revealed connections from neighboring tonotopic locations targeted to L2/3 neurons from deeper layers (Oviedo et al., 2010). Nearby intracortical inputs within L2/3 appear to have an anisotropic organization such that there is a greater spatial range of excitatory connections across the tonotopic map, arising from other L2/3 cells representing different frequencies (Watkins et al., 2014) compared to connections within isofrequency regions. Together these observations suggest the existence of cross-tonotopic convergence onto cells in the upper layers of A1.

Similarly, there is evidence that layer 4 (L4) neurons receive convergent cross-frequency inputs, although their thalamocortical inputs are narrowly tuned. The thalamocortical input from the ventral division of the medial geniculate body (MGBv), which represents the lemniscal pathway, is targeted in a tonotopic manner to L4 and to some extent, layer 3 (L3), cells (Velenovsky et al., 2003; Hackett et al., 2011). The thalamocortical synapses have a particularly strong influence on L4 cells (Liu et al., 2007; Lien and Scanziani, 2013) by virtue of ending on proximal dendrites and having high release probability (Rose and Metherate, 2005; Liu et al., 2007; Richardson et al., 2009). However, based on studies in visual cortex, thalamocortical synapses are thought to only account for ~5% of the total number of synapses onto L4 neurons (Douglas and Martin, 2007a), with the remaining 95% of the synapses originating from intracortical and other sources. Notably, many L4 cells in A1 differ from the stellate cells in visual or somatosensory cortex because they have an apical dendrite that extends into L2/3 (Smith and Populin, 2001). L4 neurons also are known to receive inputs from L6 (Lee and Sherman, 2008, 2009), although the spatial organization of these infragranular inputs is not clear.

To examine the spatial organization of intracortical inputs to L4 neurons in A1, we used laser-scanning photostimulation (LSPS) with glutamate uncaging (Callaway and Katz, 1993) to excite cortical neurons and measured synaptic responses in L4 neurons. Our results show that although the spatial pattern of intracortical inputs to individual L4 neurons is variable, a local synaptic input from L4 cells within 100 μm is a consistent feature. Other common features of the input maps include connections from L4 and L6 neurons in isolated regions 300–500 μm rostral or caudal to the recorded cell, possibly corresponding to cells tuned to different sound frequencies, and a set of vertically oriented inputs from L2 through L6. Thus, L4 cells are the target of intracortical circuits that may allow them to participate in the spectral integration of the acoustic environment at the earliest stages of auditory cortical processing.

## Materials and methods

All experiments used CBA/CaJ mice (Jackson Labs) from in-house colonies that were 35–43 days postnatal (p35–43). All animal use followed a protocol approved by the University of North Carolina Institutional Animal Care and Use Committee.

### Dissection

Mice were anesthetized with an intraperitoneal injection of 100 mg/kg Ketamine and 10 mg/kg Xylazine. After the mice became areflexic, they were decapitated, and the brain was removed and immersed in ice-cold dissection solution. The dissection solution contained (in mM): N-methyl-D-glucamine 135, choline-Cl 20, KCl 2.2, KH_2_PO_4_ 1.2, NaHCO_3_ 20, glucose 10, MgSO_4_ 1.5, CaCl_2_ 0.5. The pH of the dissection solution was adjusted to 7.4 with HCl. The brain was trimmed ~2 mm rostral to auditory cortex, caudally at the level of the midbrain, and bisected along the midline. Pieces containing auditory cortex were mounted to an angled block using cyanoacrylate glue, such that slices of the cortex were taken at an angle of 15° above horizontal (Cruikshank et al., 2002). In this plane of section the rostral-caudal dimension contains neurons from across the tonotopic map. Slices (400 μm thick) were cut using a Leica VT1200 oscillating slicer, and transferred to a holding chamber containing an artificial cerebrospinal fluid (ACSF). The ACSF contained (in mM): NaCl 122, KCl 1.75, KH_2_PO_4_ 1.25, NaHCO_3_ 25, glucose 10, myo-inositol 3, Na pyruvate 2, ascorbic acid 0.4, MgSO_4_ 4, and CaCl_2_ 4. This solution was gassed with 95%O_2_-5%CO_2_, and had a pH of 7.2–7.3. The slices were placed in the holding chamber at 34°C for 15–20 min, after which the chamber was allowed to cool to room temperature. Slices remained in this chamber for 30 min to 6 h until they were transferred to the recording chamber. The time between decapitation and transfer of slices to the holding chamber was 15–20 min.

### Electrophysiology

Slices were secured in a 0.3 mL recording chamber supplied with a 5 mL recirculating bath containing gassed (95% O_2_-5% CO_2_) ACSF supplemented with 250 μM MNI-caged glutamate, 500 μM (S)-α-Methyl-4-carboxyphenylglycine (S-MCPG) and 50 μM D-2,5-aminophosphonovalerate (D-APV). S-MCPG was used to reduce the activation of group I and group II metabotropic glutamate receptors and potential initiation of apoptosis (Boucsein et al., 2005) from build-up of glutamate that had been uncaged during the course of the experiment. D-APV was used to minimize polysynaptic excitation. Calcium and magnesium concentrations were elevated to 4 mM each to increase synaptic release probability, and raise action potential thresholds (Shepherd et al., 2003; Jin et al., 2006). Together, these conditions permit a more reliable detection of connections than standard slice solutions, while also minimizing the likelihood of activating polysynaptic circuits. The bathing solution was pumped through the recording chamber at ~2 mL/min. All recordings were performed at room temperature (20–25°C). We visualized cells for patching with a Zeiss Axioskop FS 2 using a 63 × 0.9 NA water-immersion objective and a CCD camera (Photometrics Quantix 57 or QuantEM 512SC).

The UV uncaging of glutamate excites cells, but the spatial extent of that excitation and the number of spikes elicited depend on the size of the laser spot, the total energy of the light pulse, and the amount and spatial distribution of glutamate that becomes uncaged. In order to understand how the glutamate uncaging excites cells, the responses to uncaging as a function of spot position and flash energy (“excitation profiles”) were measured in cell-attached or whole-cell current-clamp mode. For these experiments, patch electrodes were pulled from 1.2 mm borosilicate glass and contained (in mM): K gluconate 130, NaCl 4, HEPES 10, EGTA 0.2, creatine phosphate (Tris salt) 10, MgATP 4, and GTP (Tris salt) 0.3. Voltage and current were recorded with a MultiClamp 700A amplifier (Molecular Devices), low-pass filtered at 2.4 kHz, digitized at 400 kHz, and down-sampled 40x, to reduce extraneous high-frequency noise, to a final sample rate of 10 kHz.

L4 neurons were recorded in voltage-clamp mode using pipettes containing (in mM): CsMeSO_4_ 128, CsCl 2, EGTA 5, HEPES 10, MgATP 4, GTP (TRIS salt) 0.3, creatine phosphate (Tris salt) 10, and QX314 Cl 3. Pipettes were pulled from 1.5 mm KG-33 glass and coated with Sylgard 184 (Dow Corning, Midland, MI). Pipette resistances were 2–4 MΩ, and cells were voltage-clamped at −70 mV to minimize contamination by inhibitory currents. Signal processing for the mapping experiments was the same as for measuring excitation profiles, except that digitized data was downsampled by 20x to a final sample rate of 20 kHz.

For all experiments, the electrode solutions also contained ~100 μM Alexa Fluor 488 or 568, or in some cases Lucifer Yellow (Invitrogen) to allow morphological identification of cells. Cells were imaged with a CCD camera, while illuminating the slice with 470 or 530 nm LED, using standard epifluorescence optics, or in some experiments with a custom multiphoton system. The morphology was used to confirm that recorded cells used for input mapping had cell bodies located in L4.

For mapping, we recorded from L4 neurons located within 510 μm horizontally of a tangent line drawn from the rostral pole of the hippocampus to the pial surface (mean distance, 63 ± 237(SD) μm caudal to the tangent). This places our recording area in the anterior region of A1 (Cruikshank et al., 2002; Broicher et al., 2010).

### Photostimulation

We used laser-scanning uncaging of glutamate (Callaway and Katz, 1993; Campagnola and Manis, 2014) to map sources of synaptic inputs to L4 cells. In our system, the laser, optics, and microscope were fixed in position, while the slice chamber and manipulators were on a motorized XY translation stage. UV stimulation was produced by a 100 mW, 355 nm diode-pumped solid-state laser (DPSS Lasers, Santa Clara, CA). The laser power was monitored with a beam sampler that reflected a small portion of the excitation light to a calibrated photodiode. The laser power at the sample plane was measured before each experiment using a Newport 1917-R power meter and 818P-015-17W thermopile sensor (Newport). The size of the stimulation spot at the sample plane was selected by adjusting the beam divergence using a telescope consisting of two 75 mm UV-antireflective lenses. The 1/e^2^ width of the Gaussian profile of the laser spot at the surface of the tissue in all experiments was 50.1 ± 3.2 μm. Following the telescope, two fixed mirrors directed the beam onto a pair of front-surface galvanometer-controlled scan mirrors (6210H, Cambridge Technologies, MA). The scan mirrors adjusted the angle of the beam through a fixed point at the back of the microscope objective to control the uncaging location, after passing through a standard scan lens configuration.

The laser output was controlled by a Q-switch, and blocked with a fast mechanical shutter (Uniblitz, Vincent Associates) to reduce leakage. The acquisition software adjusted the duration of the laser Q-switch activation to regulate the amount of stimulus energy that reached the sample. For excitation profiles, a range of energies from 1 to 7 μJoules (μJ) per pulse was used. For all mapping experiments, the total energy per pulse was set to 5 μJ.

All data were acquired using ACQ4 (Campagnola et al., 2014), available at www.acq4.org. ACQ4 coordinated the photostimulation, scan mirror commands, physiological recording, and camera imaging during experiments. Devices were synchronized with a multifunction 16-bit data acquisition card (National Instruments PCI-6259) controlled by ACQ4. Scan mirrors were automatically calibrated prior to experiments by registering the position of fluorescence produced in a sample by the laser spot against an image on the CCD camera. Thus, during experiments, stimulation sites were specified by visually aligning the desired scanning pattern relative to an image of the slice, and the positions of flashes on each slice could be verified by capturing UV-induced fluorescence during each flash.

Stimulation sites were spaced 35 μm apart in a hexagonal grid over auditory cortex. In order to map an area larger than the field of the 5X objective lens, multiple sets of smaller maps were combined. By recording the position of the XY stage, multiple maps obtained over different cortical regions after translation of the slice could be precisely aligned during analysis, and registered against a mosaic image of the overall slice. For each mapped cell, the stimulation sites extended from L2 to L6 (and occasionally into the white matter), and at least 450 μm rostral and caudal to the recorded cell. The average lateral extent of stimulation was 765 μm caudal to the cell and 725 μm rostral to the cell. The region occluded by the recording pipette was excluded from the stimulation grid to prevent spatial artifacts that could be introduced by refraction of the laser beam through the glass walls of the pipette. In the standard orientation of the slice in the chamber, this area included parts of L1 (which was not mapped) and a sliver of L2/3 between the cell and the pial surface. As a result, vertical connections between L2/3 and L4 are likely underrepresented.

### Event detection

Excitatory post synaptic currents (EPSCs) were low-pass filtered and exponentially deconvolved (Richardson and Silberberg, 2008), and a threshold was used to detect the onset time of the deconvolved EPSCs. These EPSCs were then re-convolved to reconstruct the original shape, fit to the sum of two exponentials to capture the rising and falling phases, and accepted or rejected based on their shape. EPSCs were accepted if they were greater than 8 pA in amplitude, had a decay time constant between 0.2 and 60 ms, and if the fractional error of the fit (calculated as the standard deviation of the difference between the EPSC and the fit, divided by the standard deviation of the fit) was less than 0.5. EPSCs that passed this detection stage were used to construct synaptic input maps. Across all cells, the 10–90% percentile of the EPSC decay time constant distribution was 1.37–10.1 ms, with a median value of 3.39 ms. Variation in the series resistance, cell input resistance and noise level across cells required that the parameters for the exponential deconvolution and threshold detection be tailored for each cell.

### Spatial correlation algorithm

Spontaneous EPSCs can confound the detection of evoked responses. To minimize the misidentification of input sites due to spontaneous events, a spatial correlation algorithm (Bendels et al., 2010) was used to determine the probability that events detected in the post-stimulus time window were spontaneous (**Figure 3**), and such events were excluded. The spatial correlation algorithm uses the probability, *p*, of observing a spontaneous event in a given time window, Δt, calculated by:

(1) $$p=1-e^{\left(-r_{s}*\Delta t\right)}$$

where *r_s_* is the rate of spontaneous events. In our analysis, Δt was 50 ms. The value of *r_s_* was determined from the baseline prior to stimulation averaged over all maps for each cell. This baseline duration varied between 100, 300, and 500 ms in different experiments. Shorter times were used in later experiments as we attempted to decrease the time needed for each map and increase the number of maps per cell. The measured probability, *p*, of a spontaneous event occurring during the post-stimulus detection window for cells in this study ranged from 0.01 (for the cell with the lowest spontaneous event rate) to 0.77 (for the cell with the highest spontaneous event rate). For the cell with the median spontaneous event rate, *p* was 0.25.

Each map was repeated 1–3 times. We also used an oversampling approach in which the stimulation sites in each map were partially overlapped. The sites were spaced 35 μm apart, which is less than both the laser beam 1/e^2^ width (~50 μm), and the average radius of the excitation profiles of L2/3 cells (45 μm, see Results-Excitation Profiles). Consequently, each neuron within the mapped area was visited between 4 and 7 times per map repetition, with sufficiently strong stimulation that the neuron would have more than a 50% chance of firing. These conditions were not met for the outer most boundaries of the map, however. Because adjacent stimulation sites activate overlapping populations of cells, stimulus-evoked events should exhibit a local spatial correlation. The probability, *F*, of observing *n* or more sites with spontaneous events in the *N* stimulation sites within a 90 μm radius of any given site was then calculated as (Bendels et al., 2010):

(2) $$F\left(n\right)=\sum_{j\;\geq \;n}\frac{p^{j}{\left(1-p\right)}^{N\;-\;j}N!}{j!\left(N-j\right)!}$$

where *n* is the number of sites with detected events, and *p* is the probability that an event is spontaneous (Equation 1). Sites where *F* < 0.01 were considered as candidate locations where cells were presynaptic to the recorded L4 cell, and this criterion level provides a stringent rejection of putative sites that arise from spontaneous events. Because this algorithm combines responses over a number of adjacent sites, it reduces the spatial resolution of the maps. However, it also increases confidence in the global structure of the maps.

### Laminar boundaries

Layer boundaries were determined visually from images of slices taken under low magnification. The boundary between L1 and L2/3 was readily identified, as was the boundary between L2/3 and L4. We then halved the distance between bottom of L4 and bottom of L6, to define the L5-6 boundary, because it was not distinct. Boundaries were measured this way in 5 slices, and the values averaged and normalized. These distances (in percentages from the pial surface) are: L1-2/3 boundary: 13%; L2/3-L4: 31%; L4-L5: 45%, L5-L6: 72%. The 100% distance corresponds to 1.12 mm, and was used to normalize depths across slices, as described next.

### Map normalization

Two factors complicate the geometric comparisons of input maps between different cells. First, the overall shape of cortex differs between slices as the depth of the cortex increases from the caudal to rostral extent of A1. Second, in some slices, there was also a slight curvature of the layers over the area that was mapped. Thus, to directly compare the maps, we transformed the positions of stimulation sites onto a normalized coordinate system. In the normalized coordinates, the x-position of each stimulation site was determined by the rostro-caudal position of the site relative to the cell after adjusting for the curvature of the cortex. The y-position of each site was scaled to the distance between the pial surface and the lower boundary of L6. We then normalized the y-positions as a percentage of the average depth across all slices (1.12 mm), so that the x and y coordinates have the same units.

To re-map the positions of cells and their associated stimulus sites onto a normalized coordinate system, the mapped area was first divided into adjacent quadrilaterals. Two sides of each quadrilateral extended radially through cortex and were orthogonal to the pial surface at their lateral-most position. The other two sides were tangent to the pial surface and the boundary between L6 and the white matter, respectively. We then solved the bilinear transform that mapped each quadrilateral to a rectangle with horizontal pial and L6-white matter boundaries and orthogonal radial boundaries (using scipy.linalg.solve from the Python library scipy, www.scipy.org). The original position of each stimulation point was then re-mapped to its normalized position using this bilinear transform.

### Fitting laminar profiles

The inputs to most cells appeared to be organized in independent clusters. To compare the spatial organization of inputs on a layer-by-layer basis, we reduced each cluster to a single point along the horizontal axis. First, we extracted the distribution of inputs from each cell by layer, compressing all detected inputs within the layer into a single representation of the probability that an input was seen at a particular horizontal distance away from the cell. This distribution was then normalized to the maximum probability, and thresholded at 15% of that maximum input probability to remove weak inputs. The resulting profile was fit with multiple flat-topped Gaussians to identify clusters, as described by Equation 3:

(3) $$\begin{array}{l} A\left(x_{x\;>\;(x_{0}\;+\;w)}\right)=\;\frac{a}{\sqrt{\sigma *2\pi }}\exp\left(\frac{-(x-{\left(x_{0}+w\right)}^{2}}{2*\sigma^{2}}\right),\;\\ A\left(x_{x\;<\;(x_{0}\;-\;w)}\right)=\;\frac{a}{\sqrt{\sigma *2\pi }}\exp\left(\frac{-(x-{\left(x_{0}-w\right)}^{2}}{2*\sigma^{2}}\right)\\ A\left(x_{x\leq \left|w\;-\;x_{0}\right|}\right)=a \end{array}$$

where *x* is the horizontal distance from the cell, *x_0_* is the position of the input, *w* is the half-width of the flat-topped region, *a* is the amplitude of the Gaussian, and σ is the half-width of the Gaussian. Peaks were identified by a simple search and defined by transitions across a threshold. The center of mass of each peak was then computed, and the peak was fit to Equation 3 with the constraint that the center of the flat-topped Gaussian be within ± 5% of the center of mass of each peak. Dips in the probability distribution between peaks that did not fall below half the mean peak height of the two peaks resulted in coalesced peak and were treated as a single cluster. The resulting center of mass of the Gaussian approximation was then used to define the position of each cluster.

### Statistical analysis

A two-sample permutation test using asymptotic approximation in R (http://www.r-project.org) was used to test whether the shapes of pre- and post-stimulus populations of events differed. Resampling tests were used to test the probability that presynaptic areas similar in size to those revealed with the mapping could arise from spontaneous activity or from evoked responses, by shuffling the positions. A resampling test was also used to estimate the 95% confidence limit for the locations of detected spots as a function of horizontal position. These last two tests are described in more detail the Results section. All values are expressed as mean ± standard deviation.

## Results

We used LSPS with glutamate uncaging to map the spatial location of cortical neurons that are presynaptic to L4 neurons in A1. Recordings were made from individual L4 neurons (22 cells in 18 slices from 15 mice) while focally uncaging glutamate systematically over the slice to stimulate potential presynaptic neurons. Whether stimulated neurons are presynaptic was determined by recording the presence or absence of excitatory post-synaptic currents (EPSCs) in the target neuron, and calculating the probability of observing spatially patterned responses given the level of spontaneous activity.

### Excitation profiles

**Figure 1:**
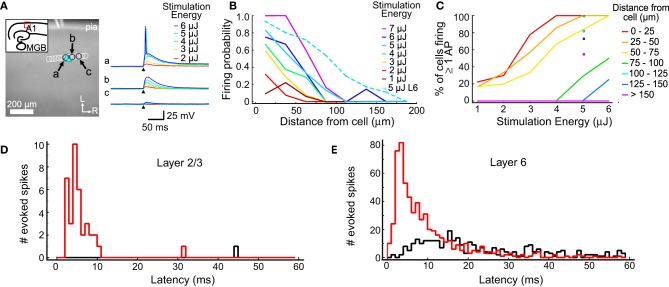
**Excitation profiles of L2/3 and L6 cells. (A)** An example excitation profile experiment. The L2/3 cell is marked by the blue triangle. Each circle represents the location of a set of stimulations at varying intensity. Traces in response to different stimulus energies (right) are shown for sites indicated by letters (a,b,c). **(B)** Firing probability for sites across all experiments (in 11 L2/3 cells and 11 L6 cells). Sites were sorted into 25 μm bins based on their distance from the recorded cell. The firing probability was high nearest the cell and decreased smoothly with increasing distance. **(C)** The percent of cells that fired at least one action potential from stimulations at the corresponding distance and intensity. Binning is same as in **(B)**. **(D)** Summary of the latency of evoked action potentials across all trials in all L2/3 cells. The red histogram shows first spike latencies; the black histogram shows latencies of any additional spikes. **(E)** Summary of the latency of evoked action potentials across all trials for all L6 cells. Histograms are color coded as in **(D)**.

In order to determine the optimal stimulation parameters, we recorded excitation profiles from cells in L2/3 and L6. In these experiments we used either cell-attached or whole-cell current clamp modes to record the firing of a cell in response to photostimulation with multiple intensities at multiple locations relative to the cell. Thus, excitation profiles measure the firing responses of neurons, as a function of the location and intensity of stimulation (Figure 1). We recorded excitation profiles from 11 L2/3 neurons at multiple stimulus intensities (in 5 slices from 4 mice; 3 cells were mapped in cell-attached mode only; 2 in cell-attached and whole-cell; and 5 in whole-cell mode only), and from 11 L6 neurons (in 3 slices from 3 mice; 5 cells were mapped in cell-attached mode only; 2 in cell-attached and whole-cell; and 4 in whole-cell mode only) at 5 μJ, the intensity we chose to use for mapping. In L2/3, 2/11 cells were electrophysiologically characterized, and both were regular spiking cells. In L6, 6/11 cells were electrophysiologically characterized. Three cells were regular spiking with slow adaptation (adaptation index < 3, computed as the mean of the last 2 interpsike intervals divided by the first interspike interval), two were rapidly adapting at the onset of the current pulse, with adaptation indices > 6, and one exhibited only a brief burst of spikes at the onset.

One goal of these measurements is to determine a stimulation intensity that reliably causes neurons to fire action potentials when the stimulation is near the cell body, but without driving action potentials from other locations, such as dendrites. The stimulation must be reliable so synaptic connections can be identified when they are present. At the same time, it is important to limit the stimulation area to improve spatial resolution and minimize evoked polysynaptic activity in the slice. Another goal of these measurements is to understand the spatial distribution of stimulus sites that could drive action potentials in putative presynaptic cells. This profile was used to guide the selection of spot spacing that was used for mapping such that individual presynaptic cells can be stimulated multiple times in a single map.

The results of the excitation profile experiments are summarized in Figure 1. Figure 1A shows the mapping arrangement for a L2/3 cell, where a series of spots were tested for their ability to generate action potentials. Traces from 3 locations (the soma; Figure 1Aa, and two adjacent sites, Figures 1Ab,Ac) are shown in the right panel, for different stimulus energy levels. Only the highest stimulus levels evoked action potentials in this cell, and then only when the stimulus spots were directly over the soma. At these levels, all stimuli not directly over the soma produced only subthreshold depolarization. The distances between the stimulation locations and each cell were measured and collected into 25 μm bins (Figure 1B). For each combination of distance and stimulus intensity, we measured the fraction of stimuli that evoked an action potential. For each stimulus intensity, the firing probability is highest near the cell body and drops off as the stimulus moves further away. For these L2/3 cells, 5 μJ stimuli resulted in action potentials for more than 80% of test sites that were less than 25 μm from the cell, but only for 30% of the stimuli 50 to 75 μm from the cell. At 5 μJ stimulation every cell we tested responded to at least one stimulus that was centered closer than 25 μm from the soma, and at least one stimulus that was 25–50 μm away. These results suggested that 5 μJ provided the best trade-off between reliable and spatially restricted stimulation, and therefore this stimulus energy was used in all mapping experiments.

Excitation profiles using 5 μJ stimulation were also performed on L6 cells, after our mapping experiments determined that L6 is a significant source of input to L4 cells. A higher fraction of stimuli evoked firing in L6 cells at all distances compared to L2/3 cells (Figure 1B, dotted line). This is most likely caused by differences in the spatial sampling. When testing excitability, L2/3 cells were stimulated with a row of sites approximately parallel to the pial surface (Figure 1A), whereas L6 cells were tested with a grid of sites covering surrounding the cell (not shown). This means that for 5 μJ stimulation there were many more sites tested in L6 cells than in L2/3 cells, and that for L6 cells, sites covered a larger extent of their dendritic trees. The firing probability for L6 cells dropped to 0.5 when the laser was 80 μm from the soma center. Figure 1C shows the firing probability as a function of stimulus energy for L2/3 cells, and at 5 μJ for L6 cells. Laser flashes at 5 μJ resulted in a high probability of spiking in both cell populations while keeping the response spatially localized.

The direct responses to laser stimulation were also used to determine the optimal analysis window to use when detecting postsynaptic responses (Figures 1D,E). The first action potential evoked by 5 μJ flashes occurred within 55 ms 100% of the time in L2/3 cells, and 98.8% of the time in L6 cells. In L2/3 neurons, the median latency of the first action potential was 4.7 ms, with a 10–90% range of 2.7–8.8 ms (Figure 1D). In L6 neurons, the median latency of the first evoked action potential was 6.3 ms and a 10–90% range of 2.3–21.3 ms (Figure 1E). Spontaneous firing was seen at only 1 out of 104 sites (of which 38 produced at least one spike) in 11 L2/3 cells, and at 3 out of 2850 sites (of which 610 produced at least one spike) in 11 L6 cells. Laser flashes at 5 μJ evoked more than one action potential in only 2.6% of sites over L2/3 cells and in 7.3% of sites over L6 cells (Figures 1D,E; black histogram). Because the majority of elicited spikes occurred with 55 ms of the stimulus, we used this as the cutoff window for postsynaptic event detection in subsequent analyses.

### Analysis of mapping data

**Figure 2:**
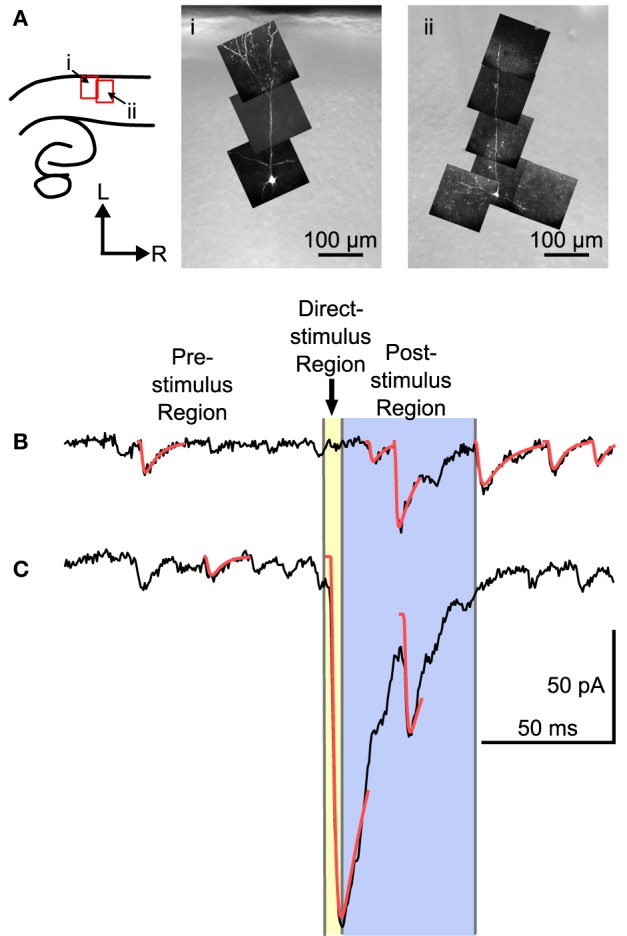
**Examples of two L4 cells and traces from a mapping experiment showing separation of spontaneous, direct, and evoked postsynaptic events. (A)** Left: The location of the images on the right shown in reference to a cartoon drawing of the slices. Right: Maximum intensity projections of multiphoton image stacks from two morphologically identified L4 cells used in the mapping study. Filled cells were spiny, with a basal dendritic tree, and, when visible, a single apical dendrite that ascended into L2/3 with or without superficial branches. **(B)** Trace showing a probable stimulus-evoked response. **(C)** Trace that shows a direct response, combined with an evoked response. For analysis, three regions were defined in each trace. The pre-stimulus region included all of the time before the stimulus. Events in the pre-stimulus region were considered spontaneous; 2 such events are shown in **(B,C)**. The direct-stimulus region (colored yellow) included the 5 ms before (to include currents caused by opening of the shutter prior to activation of the Q-switch) and after the stimulus. Events that started in this region were presumed to have been caused by uncaged glutamate binding directly to the recorded cell. The post-stimulus region was from 5 to 55 ms after the stimulus (colored blue), and events that started in this region were included in further analysis as putative postsynaptic responses. Arrow-head indicates time of laser stimulus. Red lines are calculated fits to the detected events.

Mapping experiments were carried out by measuring the synaptic response evoked by UV laser flashes at sites arranged in a hexagonal grid while recording from single L4 neurons. L4 neurons were identified according to position of the cell body (*N* = 22), and were further identified according to morphology when viewed in fluorescence, and reconstructed *in situ* either using 2-photon microscopy (*N* = 7), or with an image stack using a CCD camera (*N* = 6). Four cells had a filled cell body but no additional morphology, and five were identified by recording electrode position only. Examples of two L4 neurons are shown in Figure 2A. These cells show a characteristic set of basal dendrites principally restricted to L4, and an apical dendrite that in a few cases reached L1 (see also Smith and Populin, 2001).

The responses to laser flashes in L4 neurons consisted of a mixture of spontaneous events, direct responses, and evoked events, as illustrated in Figures 2B,C. The synaptic currents were detected (see Materials and Methods) and sorted into three categories based on the latency of the event. The pre-stimulus category included events that occurred before the laser stimulus (Figures 2B,C). These events were presumed to be spontaneous, and could include both miniature EPSCs and EPSCs arising from other cells in the slice that were spontaneously firing. The direct category includes currents produced when the uncaged glutamate binds directly to receptors on the recorded cell. Direct responses were defined as events that began less than 5 ms after the stimulus (Figure 2C). The post-stimulus category included events that began between 5 and 55 ms after the stimulus (Figure 2B). Events in the post-stimulus detection window could be either evoked EPSCs resulting from presynaptic stimulation, or spontaneous EPSCs.

Most connections between cortical neurons have more than one synapse, so the average amplitude of events in the post-stimulus window should be larger than in the pre-stimulus window. Post-stimulus EPSCs had slightly, but significantly larger average amplitudes (−15.3 ± 2.24 pA vs. −12.3 ± 2.06 pA for pre-stimulus events, *p* < 0.001, permutation test, *n* = 22 cells) and slower average decay time constants (5.6 ± 1.9 ms vs. 4.3 ± 1.2 ms for pre-stimulus events, *p* = 0.013, permutation test, *n* = 22 cells) than pre-stimulus EPSCs. The longer decay time is consistent with expectations that multiple synapses from a presynaptic cell may not release synchronously, that glutamate uncaging may generate more than one action potential in a presynaptic cell that contributes to an EPSC, and that EPSCs may also arise from asynchronous action potentials from multiple presynaptic cells. The rise times of pre- and post-stimulus events were not significantly different (1.7 ± 0.46 ms vs. 2.0 ± 0.57 ms, respectively, *p* = 0.088, permutation test, *n* = 22 cells).

**Figure 3:**
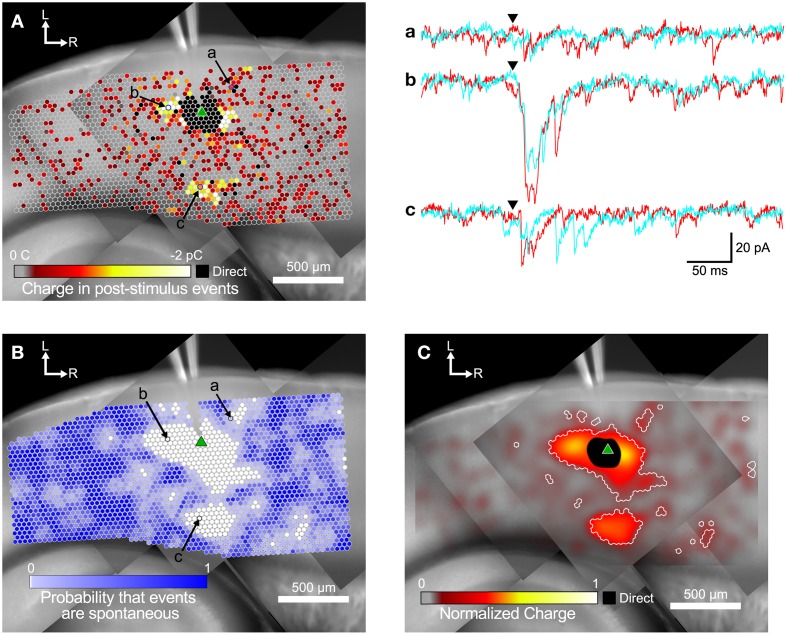
**Steps in the analysis of input maps. (A)** Color-coded maps showing detected events are superimposed over a mosaic image of the slice. The recording electrode is visible at the top center of the image. The slice was stimulated at each site (indicated by circles) while recording from a target cell (marked by green triangle). Spots where events were detected are colored according to the sum of the charge in the detected events, with hotter colors (white and yellow) indicating a larger charge. Example traces for two repeated trials at the labeled spots in **(A,B)** are shown in a, b, and c to the right. Arrowheads indicate the time of the laser flash. **(B)** The same map as in **(A)**, but spots are colored by the probability that events were spontaneous, as determined by the spatial correlation algorithm. **(C)** Same map as in **(A,B)**. Here the charge measurements are normalized and smoothed with a Gaussian convolution that has the same width (σ = 45 μm) as the excitation profiles measured in Figure 1. Areas where the probability of events being spontaneous was <0.01 are outlined in white.

To measure the spatial pattern of connectivity for each cell, two analysis steps were performed. Figure 3 illustrates the analysis processes. In the first step, we measured the total charge transfer for post-stimulus events at each position in the LSPS map, as an indication of the strength of synaptic connections. Note that this is not just the total charge of the current in the postsynaptic window, but is the summed charge of individually detected events, and so is not biased by residual slow currents from direct responses.

An example map based on the charge measure for each stimulated point is shown in Figure 3A. The small circles represent individual stimulation sites. The color of each site corresponds to the charge summed over all events in the post-stimulus time window. EPSCs appeared both in spatial clusters and as sites scattered sparsely throughout the stimulated area. The cell shown in Figure 3A has a cluster of weak inputs from L6 (Figure 3A, subset c), and stronger inputs from L4 about 300 μm caudal to the cell (Figure 3A, subset b). This cell also has a cluster of small inputs from L2/3 rostral to the cell (Figure 3A, subset a) and from L6 about 600 μm rostral to the cell. Sites near the cell show direct responses (colored black) caused by uncaged glutamate binding to receptors directly on the recorded cell. These direct responses can partially or completely mask synaptic responses from those sites. The largest direct responses arise from stimulation near the soma of the cell, but smaller direct responses were still sometimes observed up to 250 μm away, presumably due to stimulation over the cell's dendrites.

Figure 3A also shows many responses that are more sparsely and evenly distributed over the mapped area. We used the rate of spontaneous events determined from the pre-stimulus time window to calculate the probability that events in the post-stimulus time window were spontaneous rather than evoked. Spontaneous event rates ranged from 0.22 to 29.3 Hz, with a mean and standard deviation of 8.32 ± 7.23 Hz, and a median of 5.93 Hz. The cell shown in Figure 3 had a spontaneous event rate of 9.52 Hz, which means that the probability of seeing a spontaneous event in the 50 ms post-stimulus time window is 0.37 (see Equation 1). Thus, of the 3373 stimulation sites in Figure 3A, approximately 1250 sites will have at least one spontaneous event in the post-stimulus window. In this particular cell, there were 1544 sites where at least one event was detected in the post-stimulus window. Thus, it is likely that many of the isolated spots that appear to have evoked events in Figure 3A were actually produced by spontaneous events.

We used the spatial correlation of responses in nearby stimulations to determine the probability that a response was due to spontaneous activity instead of evoked by stimulation. Because LSPS sites were spaced only 35 μm apart, adjacent sites can include responses to stimulation of overlapping set of cells, and thus events from presynaptic cells should be spatially correlated. Only sites where the probability that a post-stimulus event was spontaneous was less than 0.01 were considered as functionally connected (Figure 3B). Using these more restrictive criteria produces a map that more clearly defines the areas where reliable responses were evoked. This map has 579 presynaptic sites that elicited a statistically defined postsynaptic response.

The map in Figure 3C combines the information from the representations in Figures 3A,B. Here, the sum of event charges at each stimulation site was mapped onto a rectangular image that was then convolved with a Gaussian kernel (σ = 45 μm) determined from the fit to 5 μJ L2/3 excitation profiles (cyan line in Figure 1B). This visually distributed the measurement of the strength of the input over an area with a weight corresponding to the probability that the stimulus evoked an action potential in a presynaptic cell in that area. We then defined boundaries around the presynaptic sites we considered synaptically connected to the recorded cell according to the analysis in Figure 3B, to delineate the locations of statistically significant inputs overlaid on the maps of smoothed, normalized charge. These boundaries are indicated by the white contours in Figure 3C.

### Sources of intracortical input to L4 neurons

**Figure 4:**
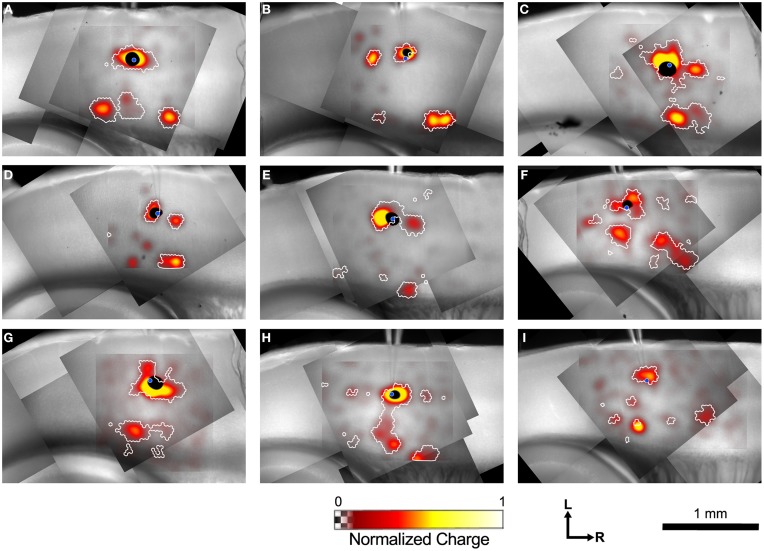
**Example input maps from 9 L4 cells. (A–I)** Each map is analyzed as described in Figure 3, and is represented in the format shown in Figure 3C. A blue circle in each map indicates the location of the patched cell. Note the discontinuous islands of input arising from locations rostral and caudal to the recorded cell.

Figure 4 shows example maps from 9 individual L4 cells, analyzed as shown in Figure 3. Common features of these maps include a local area of inputs in L4 surrounding the recorded cell, discrete patches of inputs rostrally and/or caudally in L4, L6 inputs from sites directly below the cell, and L6 inputs from sites rostral and/or caudal to the cell. Many of the maps only have a subset of these common features, whereas others have additional unique features.

Inputs from L2/3 were scattered and usually weak (Figures 4B,C,E,F) except for a few cases that arose directly from above the cell in the lower part of L2/3 (Figures 4C,F, G). In contrast strong local input from adjacent sites in L4 are visible in all maps shown in Figure 4. Additional inputs to L4 cells from L4 arise from sites further away. For example, the cell in Figure 4B has inputs from an area ~300 μm caudal to the cell body, whereas cells shown in Figures 4C–E have inputs from discrete areas rostral to the cell. Cells F and H have weak patchy L4 inputs away from the cell body as well.

Inputs to L4 from the deep layers were also prominent. Inputs from L5 to L4 that are vertically aligned with the cell are visible in the maps of Figures 4F,H (and perhaps weakly, Figure 4D). Additional inputs from slightly displaced rostral and caudal sites are also apparent (Figures 4C,F,I). Strong inputs from L6 to L4 are also evident in many maps. These may be aligned vertically (Figures 4A,C,H,I), but also appear displaced caudally (Figures 4A,B,C,H,I, also a weak input in 4E) and rostrally (Figures 4A,B,D,H,I) by 250–600 μm.

### Population average map

**Figure 5:**
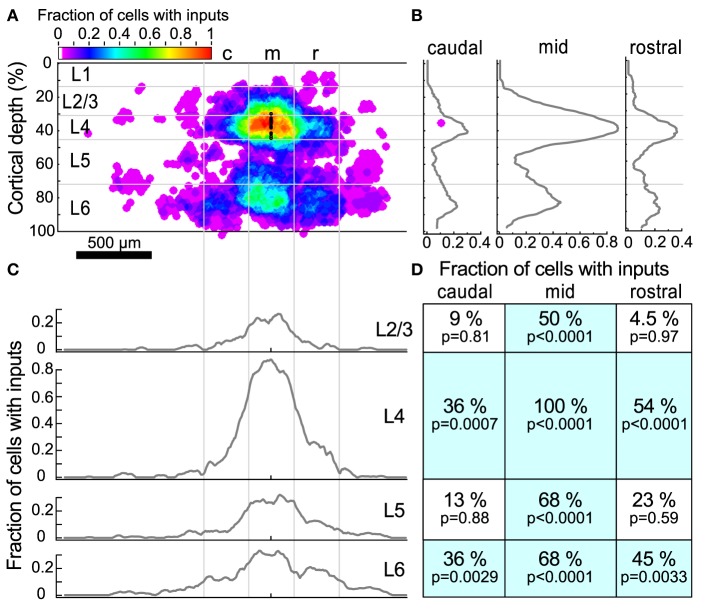
**Input maps combined across all L4 cells. (A)** The fraction of cells (*n* = 22) that had statistically probable inputs from each location. Locations were normalized onto a standardized grid as described in Materials and Methods. The map was divided into 300 μm wide columns, with the middle column centered on the recorded cell. Black dots indicate the location of patched cells. **(B)** The average fraction of cells with probable inputs across each column. Note the peaks in L4 and L6. **(C)** The average fraction of cells with probable inputs across each layer. Note the slight dips in connection probability between columns. **(D)** The fraction of cells that had a statistically probable input area larger than 10^4^ um^2^ (approximately 9 adjacent sites) within each section. *p*-values indicate the probability that the observed frequency of inputs from a given area differs from that expected from a uniform distribution of inputs (see text).

Because the input maps for L4 cells demonstrated several common features, we next combined the input probability maps across all 22 L4 cells to obtain a population overview of the spatial organization of inputs as well as the probability that inputs were observed from particular source regions (Figure 5). To do this, the maps from individual cells were first thresholded so that only statistically significant (*p* < 0.01, corresponding to the white contours in Figure 4) locations were represented. Next, the mapped locations were normalized for cortical depth, remapped onto a standard grid to account for variations in the size and shape of A1, and aligned to the L4 cell soma, as described in Materials and Methods.

The sources of inputs to L4 neurons that are visible in the maps of individual cells are also apparent in the combined map (Figure 5A). These include L4 input from cells immediately surrounding the target cell, L4 inputs from horizontal flanking regions, vertical aligned L6 input, and L6 input from horizontal flanking regions. Averaging the maps across cells obscures the detailed modular structure that is visible in individual maps because the input locations vary relative to the cell. For example, although several individual maps show clear patches of input from flanking L4 (Figures 4B,D,F,H) or L6 (Figures 4A,B,D,H) areas, in the combined map these flanking inputs appear to merge. Figures 5B,C show profiles of input site frequency averaged vertically (“columnar”) and by layer, respectively. Figure 5B shows that the L6 input is seen with about the same frequency as the L4 input in regions immediately rostral and caudal to the cell, whereas in the region aligned vertically with the cell (e.g., columnar), local L4 inputs predominate. In L4 and L6 (Figure 5C), the average profiles of inputs across the caudal-to-rostral axis lose the patchy quality of inputs that can be observed from individual maps; instead inputs appear to have a broad spread across the region, extending in some cases over 600 μm away from the cell.

To further assess the structure of individual maps while combining maps across cells, we divided the cortex around the cell into 12 rectangular regions. These 12 rectangles formed a grid with horizontal boundaries defined by layers, and three 300 μm wide “columns,” symmetrically arranged around the cell. For each region, the fraction of sites associated with an input area larger than 10^4^ μm^2^ (approximately 9 adjacent stimulation sites) was determined (Figure 5D). This analysis showed that all cells had local L4 inputs, whereas 36% of cells had caudal L4 input and 54% of cells had rostral L4 input. Although many L4 cells had inputs arising from L2/3 and L5 cells directly above and below (50 and 68% respectively), fewer cells had rostral or caudal input areas in L2/3 or L5. The horizontal offset of the rostral and caudal L6 inputs approximately matched the rostral and caudal L4 inputs. Thirty-six percent of cells had inputs from the caudal L6 region and 45% of cells had inputs from the rostral L6 region. Sixty-eight percent of cells had vertically-aligned L6 inputs.

### Reliability of the spatial analysis

**Figure 6:**
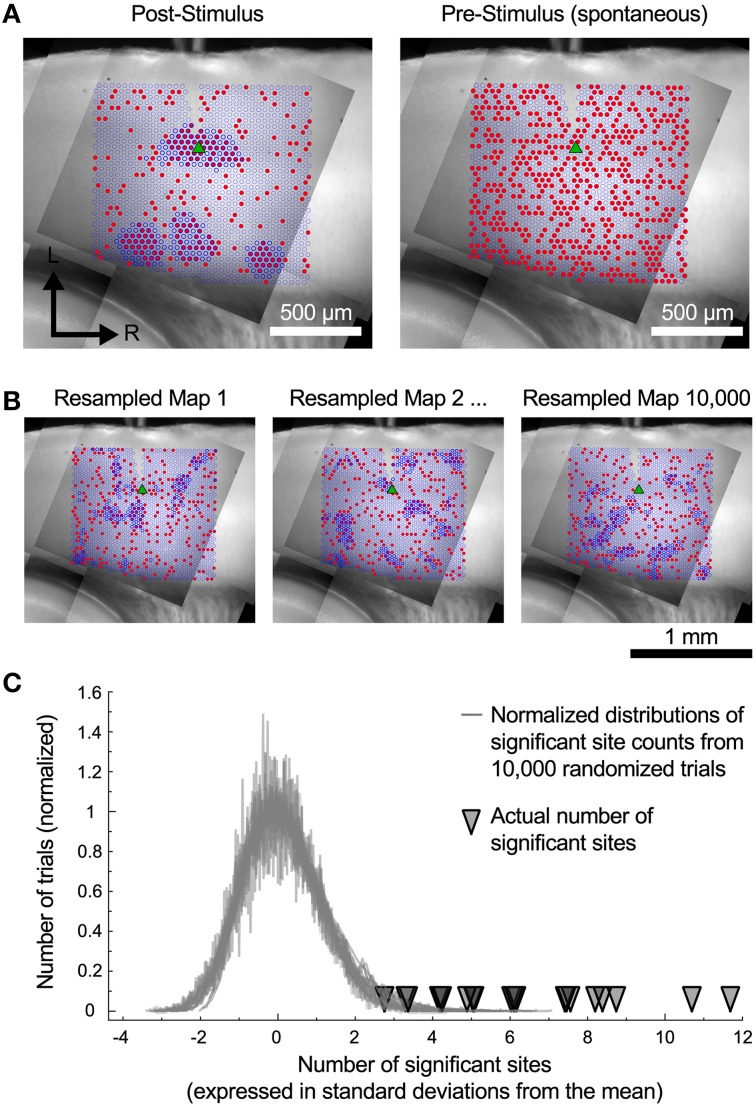
**Resampled maps. (A)** Maps of the spatial correlation of post-stimulus and pre-stimulus (spontaneous) events before random resampling. Red circles indicate sites with detected events. Dark blue outlines indicate sites detected by the spatial correlation algorithm. **(B)** Three examples of the same cell as in A with the event locations randomly shuffled. **(C)** The number of spatially correlated sites found in actual maps was always much higher than the distribution of such sites that would be detected if the spatial locations of individual inputs are shuffled.

The cells in the auditory cortex had a high rate of spontaneous synaptic activity, which led us to consider whether some of the clusters of inputs that we have observed could have resulted from chance association. To test this, we used a spatial resampling technique to examine the reliability of the spatial correlation algorithm. First, we tested the spatial correlation algorithm on the pattern of spontaneous events recorded in the pre-stimulus window. For each cell, we found either zero or very few sites with spatially correlated inputs (Figure 6A, right). This suggests that the spontaneous synaptic events alone cannot account for the results we observe, even for the smallest regions. Second, we also examined events in the postsynaptic window. For each cell, the responses associated with each stimulation site were randomly shuffled in space (Figure 6B) to generate a resampled map. We then applied the spatial correlation algorithm to the resampled maps, and counted the number of sites with spatially correlated inputs (Figure 6B). This process was repeated 10,000 times. For all cells, the number of sites that were identified as inputs in the original maps was more than 2.8 standard deviations above the mean of the number of sites identified in the resampled maps (Figure 6C). This strongly suggests that the clusters of input areas that we identified were unlikely to be an artifact arising from spontaneous events.

We next computed the probability of randomly seeing inputs with the spatial distribution we observed, using the same spatial resampling. To accomplish this we counted how many cells had input areas greater that 10^4^ μm^2^ in each region in Figure 5D. We then took the number of inputs that we actually observed in each region and calculated the probability of randomly observing the same or greater number of inputs in that region from the resampled maps. These probabilities are shown as the *p*-values in Figure 5D. This shows that the spatial clustering of individual inputs that we observed would be expected to occur very rarely in our sample if they arose only from the same frequency of events across the map without local spatial clustering.

### Horizontal spacing of inputs

**Figure 7:**
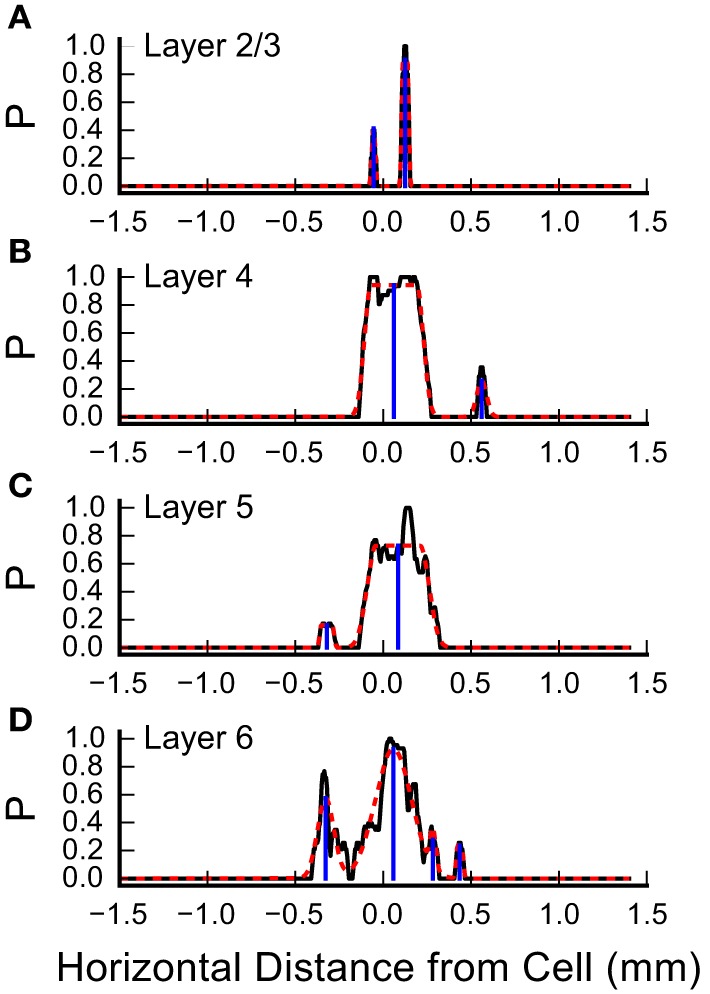
**Measurement of the center positions of input sites, by layer**. Shown are the summary probabilities of input in each layer for a single cell, as the probability of an identified input site occurring at each horizontal position, collapsed by layer, relative to the cell, plotted with solid black lines. **(A)** Layer 2/3, **(B)** Layer 4, **(C)** Layer 5, and **(D)** Layer 6. In each plot, the dashed red lines show best fits of flat-topped Gaussians (see Materials and Methods) to the probability profiles. The computed center of mass of the individual Gaussians are shown by the blue drop lines.

Inspection of the plots in Figure 4 and Figures 5A,C suggests that some of the L4 and L6 inputs arise from locations 300–500 μm rostral and caudal to the recorded cell, and in some cases these appear as discrete clusters. However, averaging the input maps across cells reveals a large central peak, with only a small peak with this spacing on the shoulder (Figure 5C). To determine whether the frequent presence of lateral clusters is due to chance or reflects a feature of the underlying input distribution, we performed an additional analysis to more precisely localize the inputs, as described in Materials and Methods. An example of this analysis from one cell is shown in Figure 7. Here, the raw profiles are plotted in black, whereas the resulting Gaussian fits are plotted with dashed red lines. Overall, the fits to the data are excellent. Fits were poorer when there are dips in the probability distribution that resulted in merged peaks, but still captured the site of the input. The center of mass of the peak along the horizontal axis was then determined (shown by the blue drop line).

**Figure 8:**
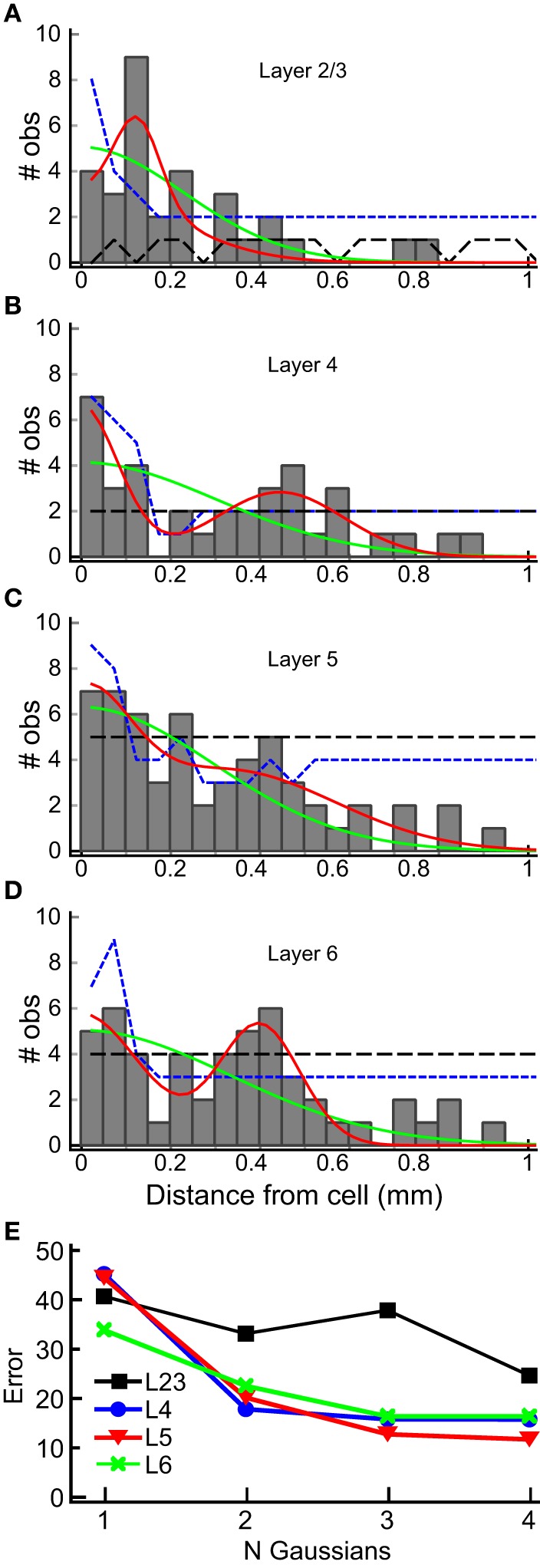
**Horizontal distribution of input sites, by layer**. The numbers of observations of center-of-mass positions computed as in Figure 7 are plotted as a function of the absolute distance from the cell body, in 50 μm bins, for each layer (gray bars) for each layer **(A–D)**. The histograms summarize combined results from all 22 L4 cells. Solid lines are superimposed plots of fits of the distribution to a single Gaussian (center position 0 μm; green line) and to the sum of two Gaussians (one with center position at 0 μm, the other position was allowed to vary). The 95% one-sided confidence interval computed by shuffling positions (5000 trials) under the assumption that the probability of a central input straddling 0 μm was the same as observed in the data set (see Results) is shown as the short dashed blue line. The 95% one-sided confidence interval computed using all input positions, and not requiring a central peak that straddles 0 μm, is shown as the long dashed black line. Note that the histogram equals or exceeds the 95% confidence interval for bins more than 250 μm away from the cell at 200, 300 and 450 μm in L2/3 **(A)**, 300 to 600 μm in L4, with the exception of 250 and 550 μm **(B)**, 200 and 450 μm in L5 **(C)**, and 200 to 450 μm, with the exception of 250 μm, in L6 **(D)**, with at least one bin falling below the 95% CI for closer distances in all cases. In L2/3, the data exceeds the 95% CI for most bins for the uniform distribution, and for intervals > 250 μm only for 300–400 μm. **(E)** Summary of error of the Gaussian fits for 1–4 Gaussians to the histograms in **(A–D)** (plots for 3 and 4 Gaussians are not shown in **A–D** for clarity). A major decrease in the fit error is seen between 1 and 2 Gaussians in L4, L5, and L6. There is little improvement with additional Gaussian terms. A lesser improvement in seen for more than 1 Gaussian in L2/3.

We used the horizontal center of mass positions to construct a histogram of the absolute value of the distance of each input patch, relative to the position of the recorded cells for each layer (Figure 8). The shapes of these population maps vary by layer. Shapiro-Wilk tests revealed that the distributions in all layers were significantly different from a normal (Gaussian) distribution (L2/3: *W* = 0.50; *p* = 1.0^*^10^−14^; Layer 4: *W* = 0.69, *p* = 2.3^*^10^−11^; L5: *W* = 0.70, *p* = 3.0^*^10^−11^, L6: *W* = 0.68, *p* = 1.39^*^10^−11^). Therefore, we fit the distribution with the sum of between 1 and 4 Gaussians, where the first Gaussian was always centered on the recorded cell body (0 mm). The results of these fits for the 1 and 2 Gaussians are shown as the overlay lines (green: 1 Gaussian; red: 2 Gaussians) in Figures 8 A–D, and the residuals from the fits for 1–4 Gaussians are shown in Figure 8E. For L2/3, the number of Gaussians had little effect on the fit error, consistent with the sparse and widely distributed input pattern (Figure 8A). The fits to L4, L5, and L6 distributions showed the largest improvement when going from 1 to 2 Gaussians (Figure 8E), and little improvement with more than 2 Gaussians. This suggests that the distribution of inputs is neither flat nor adequately represented by a single population of inputs centered on the cell.

Next, to determine whether the peaks in the histograms in Figure 8 were statistically significant, we computed the 95% confidence interval for each of the input locations using resampling techniques with two different assumptions. First, we tested whether the peaks in the spatial distribution were significantly above what would be expected for uniform spatial distribution, i.e., to test the hypothesis that the observed spatial pattern is independent of distance from the cell. The resampled data were drawn from the distribution of all detected input sites in all 22 cells for each layer, using number and widths of peaks as measured across the 22 cells from the analysis in Figure 7. We generated 5000 such spatial distributions, in which the positions of inputs were uniformly drawn from the range 0–1 mm, and recomputed the histograms to reconstruct a potential spatial distribution for each of the 5000 tests. The positive 95% confidence interval for each 50 μm wide spatial bin resulting from this test was then computed. This is shown as the black, long-dashed line in Figures 8A–D. Second, we tested the data against a distribution with the same probability of “central” inputs that span the center position of the cell (e.g., vertically aligned inputs), together with a uniform distribution of all other inputs. To do this, we shuffled the input sites and widths in the same way as above for each layer, but first drew a center peak without replacement from the distribution of center peaks (inputs that were “on column,” and so straddled 0 mm), with a probability based on the frequency of observation of center peaks in the 22 cells. This tested whether the presence of the high-probability center peaks affected the overall number of observations for each spatial bin. The results of this last computation are shown in Figures 8A–D as blue, short dashed lines. The peaks in the histogram located at distances from the cell between 300 and 600 μm in L4, 300 and 450 μm in L5, and 200 and 450 μm in L6, were at or above the 95% confidence interval.

Taken together, the Gaussian fits and resampling tests suggest that the rostral and caudal inputs to L4 cells from L4, L5, and L6 arise from discrete locations across the tonotopic map. Inputs from L2/3 did not show a clustered distribution. The inputs from L2/3 to L4 do not often arise from directly above the L4 cell, but from locations that are approximately 140 μm lateral to the somatic position. However, this is likely an artifact arising from the structure of the tested sites, since we excluded sites that were obscured by the recording electrode (see Materials and Methods), which was oriented perpendicular to the edge of the slice and crossed L1, L2 and L3 in the region directly above the L4 cell.

## Discussion

We have used LSPS with glutamate uncaging to map locations of cells presynaptic to L4 neurons in mouse A1 in slices cut to contain neurons from across the tonotopic spectrum. We found that L4 neurons in auditory cortex receive input from neurons located in spatially discontinuous regions of A1. Inputs to L4 cells include both vertically-oriented (columnar) inputs and inputs from regions horizontally displaced from the cell. All of the mapped L4 cells received input from immediately adjacent sites in L4. About 68% of L4 cells also received L6 input from a location directly beneath the cell. We had hypothesized, based on the presence of a dendrite extending into L2/3, that cross-tonotopic inputs arising from L2/3 would be prominent in L4 cells. Surprisingly, inputs from L2/3 to L4 were sparse, and the majority of detectable cross-tonotopic inputs originated in L4, L5, and L6, including from discrete, discontinuous regions from both the caudal and rostral sides of the cells. The rostro-caudal pattern of inputs in L4 and L6 supports the idea that information from different tonotopic locations converges onto single L4 neurons in A1. The spatial segregation of these inputs zones suggests the presence of modular processing circuits arising within the infragranular layers of cortex.

### Caveats

One of the strengths of using LSPS for mapping patterns of input to target cells is that fibers of passage are not activated, so that evoked responses must arise from local circuitry. In addition, with appropriate experimental conditions, the probability of observing polysynaptic activation can be minimized (for example, by using high divalent ion concentrations, and APV to block NMDA receptors, as done here). However, the method has a number of limitations that should be considered when interpreting the results. First, not all cells in the tissue will have the same thresholds for responding to glutamate uncaging. Factors affecting individual cell responses include the resting potential, voltage distance from rest to spike threshold, the density and type of glutamate receptors, and the intrinsic excitability of the cell, which contributes to the pattern of spikes produced in response to the stimulus. In addition, the spatial relationship between the cell's excitable membranes and the illumination spot influences the effectiveness of each stimulus. Consequently, it is difficult to know exactly which presynaptic cell classes are contributing to a particular input. Previous studies have demonstrated that at appropriate photostimulation levels, cells spike only when stimulated over regions close to their cell bodies (Schubert et al., 2001; Schnepel et al., 2014). However, presynaptic sites can be incorrectly identified if dendrites are electrically excitable, particularly when observing longer latency responses. However, our excitation profiles suggest that for most cells, no more than one action potential was evoked when uncaging over regions where the dendrites were densest near the cell body, and action potentials were never evoked by stimulation further than ~170 μm from the cell (Figure 1), suggesting that stimulation of more distal dendrites did not contribute to the maps we observed. (We also did not stimulate in L1 where the excitable distal dendrites of L5 pyramidal cells are located).

An additional issue is that, in these experiments, we increased spiking thresholds and synaptic release probability (P_r_) by raising extracellular divalent concentrations. Although this manipulation helps to increase the signal-to-noise ratio with respect to detection of monosynaptically-evoked inputs, and reduces activation of polysynaptic pathways (which were also limited by the presence of APV in the bath), it may also bias the detection of different types of inputs in two ways. First, input sources whose synapses would have low P_r_ with physiological divalent ion concentrations will become more detectable. Although inputs with normally high release P_r_ will also have increased P_r_, their detectability is not likely to increase as much. Thus, this manipulation may result in a distorted representation of the normal strength of different classes of inputs. Second, raising divalent concentrations may also affect the intrinsic excitability and spike thresholds of different classes of cells in different ways. Thus, although we have sampled some of the input cell types, the resulting probabilities of inputs from different sites should not be taken as a quantitative representation of the relative strengths of those inputs under physiological conditions.

It is also unclear how and whether a normalization should be introduced in the analysis to try to correct for the sensitivity of different types of cells or cells in different layers. We have chosen to present maps without such layer-by-layer corrections, using only the spatial information derived from the average excitability of L2/3 cells, and recognizing that these maps represent the patterns of input observed under our particular recording conditions. These maps may selectively over- or underrepresent the different sources of input to L4 cells. Our experiments demonstrate the existence of spatial patterns of functional connections whose role and strengths within the cortical circuit must be investigated with other approaches.

A interesting observation is the relative scarcity of L2/3 and L5 input to the L4 cells in our results. Although the L2/3 input was sparse, the LSPS stimulation was shown to definitely excite L2/3 cells under the conditions used for mapping, so it is unlikely that this result was due to differences in excitability of the cells. Similarly, L5 cells in auditory cortex have been shown to have resting potentials that are slightly depolarized relative to cells in other layers (Huggenberger et al., 2009; Krause et al., 2014), whereas the absolute spike threshold voltage is the same as cells in other layers. This suggests that L5 cells should be readily excited by the stimulation parameters used here. Either case could result if the synaptic inputs have a low conductance, lower release probability, or could reflect relatively less dense connectivity than from L4 and L6. However, these possibilities are better addressed with different techniques.

### Comparison with previous studies

Previous studies have investigated local intracortical connections in auditory cortex using both anatomical and electrophysiological methods. Ojima et al. (1992) reported axonal projections of a subset of single intracellularly labeled infragranular (L6) neurons in A1 of cats had collaterals in L3 and L4 as well as in L6. The axon collaterals with terminals in L3 and L4 extended ventrally from the L6 cell body, remaining within the isofrequency sheet, whereas the axon collaterals within L6 extended dorsally. Whether the L4 projections of these cells also cross the tonotopic axis is not clear. “Patchy” or modular projections between regions in A1 have also been observed with anterograde tracing methods. In cat A1, injections of a tracer into L5 and L6 resulted in patches of labeled axon terminals in upper layers, including L4, as well a spread of axon terminals in deeper layers. These patches were primarily contained within isofrequency sheets, but also appeared in regions with higher or lower best frequencies relative to the injection site (Wallace et al., 1991).

Barbour and Callaway (2008) used LSPS to map inputs to L2/3 and L4 cells using slices oriented orthogonal to those used here. As a result, only vertically arranged connections can be compared with their study, since this is the only axis that is common to both planes. Consistent with our results, Barbour and Calloway saw substantial local L4 input to L4 cells. They reported L2/3 input in 3 out of 13 L4 cells, which is consistent with the sparse input pattern we observe. They also reported a small amount of vertical L5 input to L4, but no L6 input. This is surprising because we saw vertical L6 input in 68% L4 cells, and vertically oriented inputs from L6 have been reported in other experiments in young (P11–18) mice (Lee et al., 2012; Lee and Imaizumi, 2013). This might reflect a species- or age-related difference; we used mice between p35 and p43, while they used younger rats, age p25–p31.

Horizontal connections similar to those we observed in L4 have also been reported onto L2/3 cells. Oviedo et al. (2010) mapped inputs to L2/3 cells in slices that were prepared both parallel and orthogonal to the tonotopic axis. They found inputs to L2 cells arose predominantly from within the same vertical position in both slice planes. They only saw columnar inputs to L3 cells in slices orthogonal to the tonotopic axis (along the isofrequency contours), but in slices cut across the tonotopic axis (the same plane of section that we used) they saw also input from L5 and L6 that originated ~250 μm rostral to the cell, which is an area that represents a higher frequency than the recorded cell. In comparison, our maps of L4 cells reveal more extensive input from cells in different regions of the tonotopic map, and arising from both rostral (higher frequency) and caudal (lower frequency) locations. Watkins et al. (2014) used a tangential slice plane to examine the spatial organization of inputs to L2/3 cells. They identified both local inputs and periodic patterns in the surrounding inputs from inhibitory neurons that were organized both orthogonal to the tonotopic axis, and within the isofrequency sheets, with approximately a 300 μm spacing. This spacing is similar to what we observe in both L4-L4 and L6-L4 connections, and suggests that these structural motifs may be present in all cortical layers, although the density of connections may vary (Figure 5).

Our results extend these observations, in that the ascending inputs from L6 to L4 show discrete patches of inputs arising from cells 300–500 μm away from the target cell. Together with other studies in mouse, these results support the existence of modular circuit interactions within the auditory cortex with spacing in the 300–500 μm range.

### Relationship of input sites to tonotopic axis

The organization of the horizontally displaced connections from L6 to L4, and their spatial discontinuity with the vertical connections, suggest that they might be part of a modular processing structure that supports interactions between cells representing different tonotopic regions, or alternatively, between cells representing similar tonotopic regions that are spatially segregated. Many of the lateral inputs in L4 and L6 arise from sites that are 300–500 μm lateral to the target L4 cell. Intriguingly, in mice, 300 μm is approximately the spacing over which the characteristic frequency of A1 neurons doubles along the tonotopic axis (Stiebler et al., 1997; Bandyopadhyay et al., 2010; Hackett et al., 2011; Guo et al., 2012), and suggests that these circuits may participate in the neural processing of sounds over at least an octave in range, or may be involved in processing sounds with harmonic structure. Many natural sounds and vocalizations exhibit harmonic structure, which arises from the resonances present in the mechanical generators of sound sources, and from non-linear acoustic processes. A substantial set of neurons in the mammalian cortex are sensitive to the harmonic structure of sounds (Wang, 2013), and this sensitivity appears to arise in cortex rather than reflecting inherited processing from lower centers. Neurons in cat primary auditory cortex often have multiple peaks in their tuning curves that are tuned to pitch features (Sutter and Schreiner, 1991), and also have an over-representation of octave-spaced response areas (Norena et al., 2008). In marmosets, neurons with multipeaked tuning curves most often have best sensitivity at frequencies that are spaced one or one-half octave apart (Kadia and Wang, 2003). In mouse, the L2/3 and L4 neurons with multipeaked tuning curves also tend to show an approximate harmonic relationship between the peaks (Winkowski and Kanold, 2013). Similarly, in mice the spatial pattern of connections in layer 2/3 exhibits a periodic structure corresponding to approximately one octave in frequency (Watkins et al., 2014). We observed intracortical neural circuits with distinct lateral connections between L4 cells, and from L5 and L6 to L4, that could support or participate in processing sounds with harmonic content, or perhaps more generally, integrating information across one to two octaves of the hearing range.

Alternatively, these patchy inputs could represent connections between cells that represent similar frequencies, but which are spatially separated. Although the tonotopic map in L4 of mouse A1 has been shown to be well organized with a regular progression of frequencies (Hackett et al., 2011; Winkowski and Kanold, 2013; Kanold et al., 2014), isofrequency contours measured in mapping experiments are not necessarily straight lines, nor do they always run parallel (Stiebler et al., 1997; Kalatsky et al., 2005). Given the orientation of our slices, it would appear that the patchy organization of inputs most likely reflects different frequencies, but with irregularities in the tonotopic map, we cannot exclude the possibility that these sites instead represent similar frequencies that are spatially segregated.

### The role of L6 inputs

In the canonical laminar distribution of inputs in the cortical circuit (Douglas and Martin, 2007b), the principal afferent input arrives at L4 from the thalamus. L4 then projects up to L2/3, which projects down to L5 and L6. L6 then sends projections back to L4. It is unclear whether this specific pattern of activity, which has been suggested based on studies in the visual cortex, also holds for auditory cortex. Recent studies suggest that the deep layer neurons in auditory and somatosensory cortex may have lower thresholds for spiking in response to thalamocortical input than L2/3 cells, and can respond earlier and more reliably than L2/3 neurons (Atencio et al., 2009; Constantinople and Bruno, 2013; Krause et al., 2014). This scheme suggests that the flow of activity includes a substantial infra-to-supragranular component, and that the deeper cells might be in a position to influence the sensory responses, even at the level of the L4 thalamocortical recipient neurons. The specific patterns of connectivity between the thalamic inputs and L5 and L6 cells, as well as the axonal organization of L5 and L6 cells, and their subsequent engagement of local inhibitory circuits then become important factors in regulating the short-latency responses of neurons in the upper layers.

There are two factors to consider in proposing roles for the L6 inputs to L4. First, the responses of L6 neurons to tonal stimuli are not uniform. Zhou et al. (2010) identified two populations of cells in auditory L6. One was excited by sound, whereas the other had a higher spontaneous firing rate that was suppressed by tones. It is not clear whether both or only one of these populations contribute to the circuits we have identified. Second, the effect of activity in L6 neurons on the upper layers of cortex appears to be primarily inhibitory. In the visual cortex, a subset of L6 cells that are defined by the expression of NTSR1-Cre contributes to a negative control of the overall gain of activity in the superficial cortical layers (Olsen et al., 2012). When this subset of L6 pyramidal cells was optically excited via channelrhodopsin, the responses of neurons in superficial layers to visual stimuli were reduced, whereas when ongoing L6 firing was suppressed via hyperpolarization with Arch-ChR2 or NpHR3.0-ChR2, superficial neurons, including L4 neurons, were more responsive to visual stimuli. Interestingly, these manipulations of L6 activity did not affect the sensory tuning of these cells. It is possible that the L6 to L4 projection has a similar functional role in auditory cortex. Lee et al. (2012) reported excitatory input to L4 cells in A1 from L6 NTSR-1 expressing cells. However, the relationships between the L6 NTSR-1 expressing cells, the two classes of L6 neurons defined by tonal stimuli, and the L6 cells that we find provide excitatory input to L4 neurons is unclear.

It has been proposed that L6 inputs to thalamocortical recipient neurons in L4 are part of a “modulator” pathway (Lee and Sherman, 2011). Connections from L6 to L4 in auditory cortex have been shown to have an mGluR component, suggesting that they contribute a modulatory input (Lee and Sherman, 2009). Our experiments support the presence of a direct, ionotropic receptor-mediated connection, but they do not exclude activation of mGluRs because we included MCPG, an antagonist of group I and II mGluRs, in the bath (see Materials and Methods). However, the strength of the L6 to L4 synaptic connections relative to those of the direct thalamocortical input is unclear.

Even if the L6 to L4 synapses are relatively weak, they still could provide a depolarizing subthreshold input to L4 cells that increases the chances that L4 cells spike in response to their thalamocortical input. Because some of the L6 cells are likely located at a different tonotopic position than their target L4 cells, this depolarization could bias the L4 cells to respond to stimuli with multiple spectral features at the tonotopic locations of both the L4 and L6 cell. Conversely, feedforward inhibition driven by L6 cells could cause suppression of responses based on such spectral features. Similar contributions might also come into play for the L4-L4 connections, which span a similar range of the tonotopic axis. Such effects would be consistent with the frequent occurrence of multipeaked tuning curves in L4 neurons (Winkowski and Kanold, 2013). The spatial arrangement of input clusters along the tonotopic axis reported here suggests a spatial relationship between underlying cortical circuits and the analysis of the spectral structure of sounds.

### Conflict of interest statement

The authors declare that the research was conducted in the absence of any commercial or financial relationships that could be construed as a potential conflict of interest.
